# Copy number variants (CNVs) analysis in a deeply phenotyped cohort of individuals with intellectual disability (ID)

**DOI:** 10.1186/1471-2350-15-82

**Published:** 2014-07-16

**Authors:** Ying Qiao, Eloi Mercier, Jila Dastan, Jane Hurlburt, Barbara McGillivray, Albert E Chudley, Sandra Farrell, Francois P Bernier, ME Suzanne Lewis, Paul Pavlidis, Evica Rajcan-Separovic

**Affiliations:** 1 Department of Pathology (Cytogenetics), BC Child and Family Research Institute, University of British Columbia (UBC), 950 West 28th, Room 3060, Vancouver, BC V5Z 4H4, Canada; 2 Department of Medical Genetics, BC Child and Family Research Institute, UBC, Vancouver, BC V6H 3N1, Canada; 3 Centre for High-throughout Biology, 177 Michael Smith Laboratories, University of British Columbia, Vancouver, BC V6T 1Z4, Canada; 4 Department of Human Genetics, University of Manitoba Children’s Hospital, Winnipeg, Manitoba R3A 1R9, Canada; 5 The Trillium Health Partners, 2200 Eglinton Avenue West, Mississauga, ON L5M 2N1, Canada; 6 Department of Medical Genetics, University of Calgary, Calgary, Alberta, Canada; 7 Department of Medical Genetics, BC Child and Family Research Institute and BC Children’s and Women’s Health Center, University of British Columbia, C234, 4500 Oak Street, Vancouver, BC V6H 3N1, Canada

**Keywords:** Intellectual disability (ID), Copy number variants (CNVs), Phenotype/genotype analysis, Clustering of phenotypes

## Abstract

**Background:**

DNA copy number variants (CNVs) are found in 15% of subjects with ID but their association with phenotypic abnormalities has been predominantly studied in smaller cohorts of subjects with detailed yet non-systematically categorized phenotypes, or larger cohorts (thousands of cases) with smaller number of generalized phenotypes.

**Methods:**

We evaluated the association of *de novo*, familial and common CNVs detected in 78 ID subjects with phenotypic abnormalities classified using the Winter-Baraitser Dysmorphology Database (WBDD) (formerly the London Dysmorphology Database). Terminology for 34 primary (coarse) and 169 secondary (fine) phenotype features were used to categorize the abnormal phenotypes and determine the prevalence of each phenotype in patients grouped by the type of CNV they had.

**Results:**

In our cohort more than 50% of cases had abnormalities in primary categories related to head (cranium, forehead, ears, eye globes, eye associated structures, nose) as well as hands and feet. The median number of primary and secondary abnormalities was 12 and 18 per subject, respectively, indicating that the cohort consisted of subjects with a high number of phenotypic abnormalities (median De Vries score for the cohort was 5). The prevalence of each phenotypic abnormality was comparable in patients with de novo or familial CNVs in comparison to those with only common CNVs, although a trend for increased frequency of cranial and forehead abnormalities was noted in subjects with rare *de novo* and familial CNVs. Two clusters of subjects were identified based on the prevalence of each fine phenotypic feature, with an average of 28.3 and 13.5 abnormal phenotypes/subject in the two clusters respectively (P < 0.05).

**Conclusions:**

Our study is a rare example of using standardized, deep morphologic phenotype clustering with phenotype/CNV correlation in a cohort of subjects with ID. The composition of the cohort inevitably influences the phenotype/genotype association, and our studies show that the influence of the *de novo* CNVs on the phenotype is less obvious in cohorts consisting of subjects with a high number of phenotypic abnormalities. The outcome of phenotype/genotype analysis also depends on the choice of phenotypes assessed and standardized phenotyping is required to minimize variability.

## Background

Intellectual disability (ID) has an overall prevalence of 1–3% [1,2] and is characterized by considerable genetic and phenotypic heterogeneity. Single gene and chromosomal disorders are considered the cause of ID in 7–37% of cases [3], while submicroscopic gains and losses (DNA copy number variants (CNVs)) occur in a further 5–15% of cases [4,5]. Screening for CNVs using chromosome microarrays is now routinely performed in subjects with ID and databases of CNVs identified in subjects with ID or controls facilitate CNV interpretation (e.g. Database of Chromosomal Imbalance and Phenotype in Human Using Ensemble Resources, DECIPHER, http://decipher.sanger.ac.uk/, or Database of Genomic Variants, DGV, http://projects.tcag.ca/variation, respectively).

The association of unique CNVs with congenital and neurodevelopmental abnormalities has been documented in reports on individual subjects, small groups of similarly affected subjects (for review see [6]) or large cohorts of patients [7-11]. Large cohort studies including thousands of cases have the benefit of assessing the overall characteristics of CNVs (e.g. size, burden) and their influence on phenotype; however, typically, they lack detailed clinical descriptions, with the phenotype derived from referral forms for array testing, rather than from a detailed chart review. Nevertheless, these studies are informative and show that large CNVs (>400 Kb) harboring more genes (i.e. large CNV burden) are more prevalent in cases with more severe developmental phenotypes associated with multiple congenital anomalies (MCA) [7], including craniofacial dysmorphology and cardiac defects, compared to ID without MCA [7,8].

Thus far, the association of the CNV presence/characteristics with a more detailed and systematic clinical description of a larger number of subjects has been rarely performed. Moreover, the various phenotypes selected for analysis mainly are based on a-priori expectations of phenotypes likely to be affected by chromosomal gain or loss. In a pioneering study, De Vries et al. investigated the association of 21 clinical features in 29 and 110 ID subjects with and without subtelomeric region copy number changes, respectively and introduced a five item checklist (i.e. de Vries Score) to help select ID patients most likely to have submicroscopic subtelomeric rearrangements (family history of ID, prenatal-onset growth retardation, postnatal growth abnormalities, ≥2 facial dysmorphic features, and congenital anomalies). Using this checklist the authors reported a significant correlation of prenatal onset of growth retardation and a positive family history with subtelomeric abnormalities [12].

In contrast, a recent study of >300 ID cases showed that pathogenic CNVs are significantly correlated with congenital heart anomalies among the 23 clinical features analyzed [13]. Prevalence of microcephaly, short stature and low weight was also higher in cases with pathogenic CNVs, but did not reach statistical significance when compared to cases without pathogenic CNVs. In our previous study of 100 cases with autism spectrum disorder (ASD) and ID [14], in which 10 major phenotypes were evaluated, we reported significant prevalence of microcephaly in cases with pathogenic CNVs and a more severe cognitive deficit in comparison to ASD/ID subjects with normal array results [14].

The most recent study correlating CNV types and phenotypes used Human Phenotype Ontology, HPO based standardized phenotyping in a cohort of >5000 ID patients [15]. However, although 34,433 HPO phenotypic features were evaluated the prevalence of only 9 “lumped” features was assesses and reported in different CNV classes (de novo, inherited and no rare CNVs). Significantly increased frequency for 7 out the 9 abnormal features was identified (Multiple congenital anomalies, Dysmorphism, Stature, Convulsions, Head circumference, Brain, Heart, Urogenital) in subjects with de novo CNVs. The patients were also assessed using a modified de Vries Score which included intellectual disability, prenatal onset of growth retardation, postnatal growth abnormalities, ≥2 dysmorphic facial features and congenital anomalies. A significant prevalence of subjects with >3 De Vries score in both the de novo and familial CNV groups in comparison to no rare CNV group was noted in their cohort which had an overall median De Vries score of 2.

Our study was designed to evaluate the association of different types of CNVs and phenotypes found in 78 patients with ID using Winter-Baraitser Dysmorphology Database (WBDD) (formerly the London Dysmorphology Database) (http://www.lmdatabases.com/about_lmd.html) and is to our knowledge the first study using this database for CNV/phenotype correlation analysis. It is also unique because the information on the prevalence of each individually detailed primary and secondary phenotype in subjects with *de novo*, familial and common CNVs was recorded, compared and reported. The patients were also clustered based on the phenotypes and the prevalence of each phenotypic feature in each cluster was assessed.

## Methods

### Subjects

A total of 78 subjects with ID were included in the analysis, recruited through a network of collaborating clinical geneticists from centers across Canada. The criteria for recruitment were based on the previously published De Vries score of 3 or higher, which resulted in enrolment of predominantly complex cases with an unknown etiology of ID. Phenotypes were collected from patient charts and confirmed by a clinical geneticist and a genetic counsellor for categorical standardization. This subset of patients was chosen based on: a) the use of array platform of similar resolution for analysis (NimbleGen and Agilent); b) availability of detailed clinical information c) previously normal karyotype and Fragile X screening. As controls we used a previously published cohort of 32 cognitively and phenotypically normal subjects (19 females and 13 males) analyzed using the same array platform [16,17]. The use of the DNA from these patients in our cohort was approved by Clinical Ethics Research Board, University of British Columbia. All subjects gave written informed consent for participation in the study and anonymized data were used for the analysis.

### Array comparative genomic hybridization (CGH)

Agilent 105 K oligonucleotide array-CGH analysis was performed according to the protocol provided by the company (version 4.0, June 2006, Agilent Technologies, CA, USA) [18]. Feature Extraction software (version 8.1.1.1, Agilent Technologies) rendered image analysis using the manufacturer’s recommended settings (CGH_v4_95) and human genome assembly hg18. The minimum absolute average of log2 ratio was 0.25. Higher-resolution 385 K oligonucleotide genome array CGH was performed by courtesy of NimbleGen. Array log2 ratio > ±0.2 was used for segmentation (region). For both the Agilent and NimbelGen array platforms, 3 consecutive probes were required for a significant CNV call. CNVs from all chromosomes were included in the analysis.

### Type of CNVs

All detected CNVs were grouped into 3 subgroups (*de novo*, familial and common CNVs) based on criterion described previously [19]. Briefly, CNVs completely overlapping with variants reported in at least two studies in the DGV or in our internal controls consisting of cognitively normal subjects [16,17] were considered common CNVs; CNVs that overlapped partially (<50%) or did not overlap with CNVs reported in the DGV or our internal controls were called unique (rare) CNVs and these included *de novo* and familial CNVs. All unique CNVs were confirmed and their origin (parental or de novo) determined by a secondary independent method (FISH or qPCR) on available cell pellet or DNA. Common CNVs from DGV v10 for hg18 have been downloaded at http://projects.tcag.ca/variation/tableview.asp?table=DGV_Content_Summary.txt. The database contained 67694 common CNVs at the time of analysis.

### Clinical feature classification

The Winter-Baraitser Dysmorphology Database (WBDD) (formerly the London Dysmorphology Database) (http://www.lmdatabases.com/about_lmd.html) was used to systematically categorize the phenotypes of each patient in our cohort. WBDD consists of 34 major clinical features as the primary category, 162 features in the secondary category and numerous further sub-classifications in the tertiary category. We used the primary and secondary categories of WBDD (named as coarse and fine phenotypes, respectively) to classify the phenotypes of our patients. We also slightly modified WBDD by adding Microcephaly and Macrocephaly as secondary categories within the Cranium-primary category (they are listed in the WBDD tertiary category). We also added the following features as separate items in the secondary category: Family history, abnormal pregnancy history, neonatal abnormality, maternal age at birth and paternal age at birth. This resulted in 169 fine phenotypic features.

For our analysis, clinical features that were present in less than 5% (i.e. in less than 4 individuals) or over 95% (i.e. in more than 74 individuals) were excluded. We eventually included 32 coarse phenotypes (after removing Neurology and Pelvis categories with 78/78 and 2/78 individuals, respectively) in the primary category and 80 fine phenotypes in the secondary category. The complete list of coarse and fine phenotypes is presented in Additional file 1: Table S1. The process of phenotype collection from chart review was extremely time-consuming, and to systematically collect the information, we used RedCap (https://cric.med.ualberta.ca/neurodevnet/) [20] for both the phenotype and CNV data storage and extraction. It not only shortened our data processing time, but also minimized any mistakes that might be induced in the process.

### Statistical analysis

#### *Computing*

All computational analysis was done using software R 2.12 for Windows (The R Project for Statistical computing: http://www.R-681project.org) [21]. Fisher’s exact test was used in comparisons of equality of proportions. CNV size comparison was performed using the Wilcoxon rank-sum test.

#### *Prevalence of clinical features in subjects with different CNV types*

Subjects were classified in groups based on the type of CNV present (*de novo*, familial or common). We computed the fraction of each abnormal phenotypic feature in these groups and tested the significance of the difference in the prevalence of each of the phenotypes between subjects with de novo versus common CNVs, and familial versus common CNVs using Fisher’s exact test (corrected for multiple tests using the Benjamini and Hochberg procedure) [22].

#### *Clustering*

We performed a k-means clustering based on a list of 80 fine clinical features. The optimal value for K (number of clusters) was chosen using the Calinski index [23], which represents the ratio of the variance within the clusters and the variance between the clusters. It is similar to an F (ANOVA) statistic. This was performed by the cascade KM function from the R package vegan 2.0–7 [24].

## Results

### Characterization of CNVs in subjects with idiopathic ID

**Figure 1:**
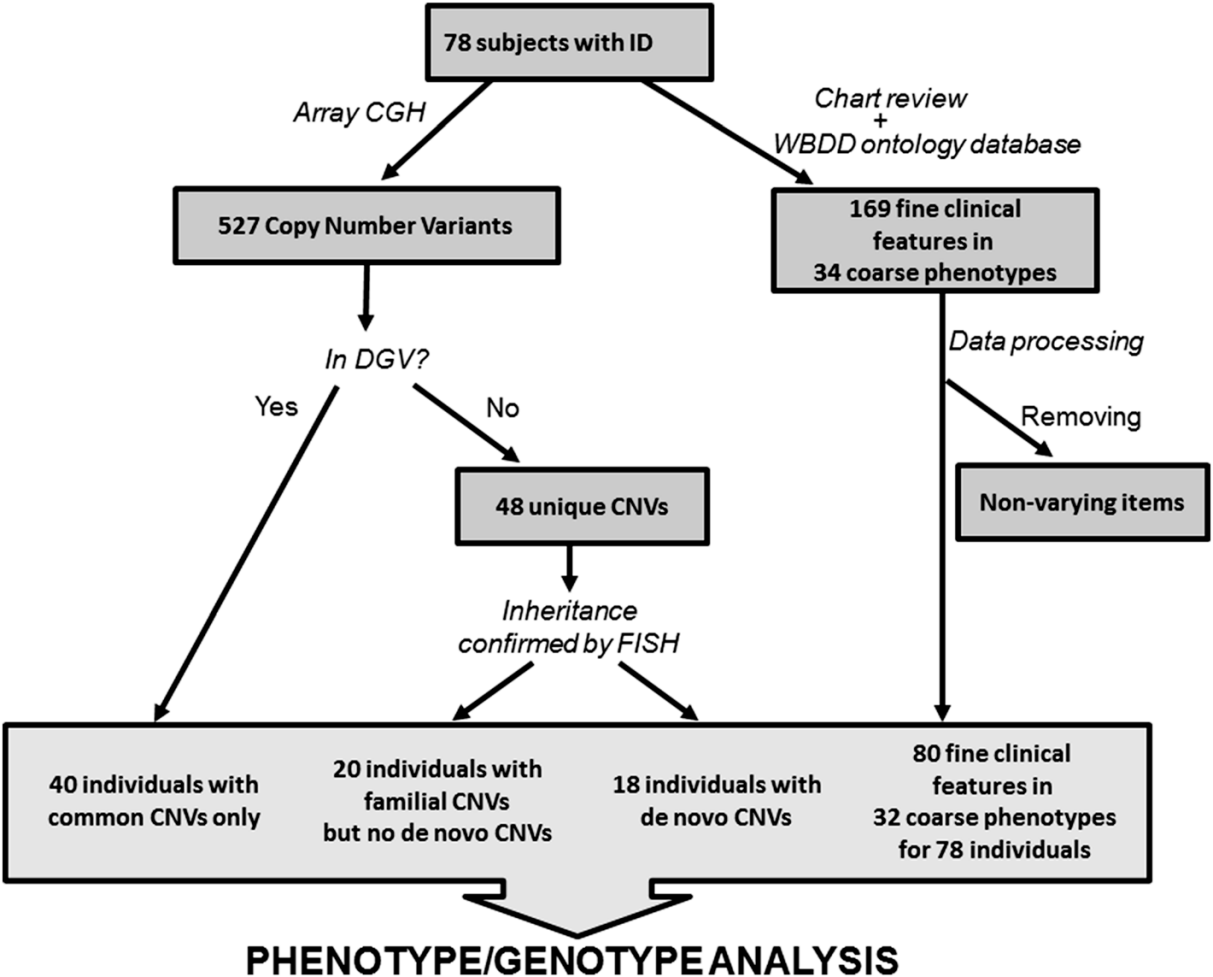
Data processing workflow.

The workflow of our study is shown in Figure 1. Using whole genome oligonucleotide microarrays (Agilent 105 K and NimbleGen 385 K), 527 CNVs were identified in 78 subjects with idiopathic ID (on average 7 CNVs/person). CNVs were classified into three subgroups based on the criteria described in Methods. Twenty-one unique *de novo* CNVs, 27 unique familial CNVs and 479 common CNVs were identified in the ID cohort (Table 1 and Additional file 1: Table S2). *De novo* CNVs ranged in size from 310 Kb to 9.7 Mb (2.5 Mb median) and were significantly larger than common CNVs (0.1 Mb median) (p = 2.3 × 10^-11^, Wilcoxon’s rank-sum test). The proportion of duplications and deletions was similar among the categories except for familial CNVs, for which 70% of cases were duplications (p = 0.002, as determined by the rank-sum test compared to pooled *de novo* and common CNVs). The proportion of deletions (and thus also duplications) in the common CNVs is similar to that observed in DGV, 59% vs. 64%, respectively. We also examined the overall gene content of the different classes of CNVs. For the purpose of our analysis, genes within 50 Kb of the estimated CNV breakpoints were included. Significantly more genes were found in *de novo* than familial or common CNVs, as would be expected based on the size difference (Table 1).

**Table 1:** CNV features comparison in different CNV types

	**Common CNVs**	**Familial CNVs**	**De novo CNVs**	**Overall for the cohort**
**Number of CNVs**	479	27	21	527
**Patients**	78	22	18	78
**CNVs/subject**	6.14	1.23	1.17	6.77
**Mean CNV size (Mb)**	0.42	0.70	3.14	0.54
**Median CNVs size (Mb)**	0.14	0.43	2.46	0.14
**Range of CNV size (Mb)**	0.01–9.68	0.03–4.36	0.31–9.78	0.01–9.78
**Genes/CNV**	5.12	7.04	33.1	6.34
**Proportion of large CNVs (>1 Mb)**	0.14	0.22	0.71	0.17
**Proportion of deletion**	0.59	0.30	0.67	0.58
**Median modified De Vries Score**	5	4.5	5	5

Six *de novo* CNVs and 5 familial CNVs overlapped with syndromic regions previously described in the DECIPHER database (Additional file 1: Table S2). Eighteen of our cases carried *de novo* CNVs (23%), with one case (5%) encompassing two independent *de novo* CNVs (2q23.3 deletion and 10q21.1 deletion) (Additional file 1: Table S2). The slightly higher prevalence of *de novo* CNVs in comparison to the literature could be the effect of enrolment criteria which was based on De Vries scoring system and typically included more phenotypically complex cases. In the unique familial CNV group, 3/22 cases (13%) have 2–3 familial CNVs. There are 2 cases having both a *de novo* and a familial CNV.

### Clinical phenotypes classification

Patient records including detailed consult letters were reviewed to categorize the clinical information in 34 coarse and 169 fine clinical features for each subject, using the Winter-Baraitser Dysmorphology Database (WBDD) (http://www.lmdatabases.com/about_lmd.htm) (Additional file 1: Table S1). The phenotypic categories were slightly modified (see Methods for details) by removing from analysis non-varying phenotypes (e.g. present or absent in more than 95% of the subjects). In addition, we included categories such as prenatal and family history (see Methods for details), and obtained a working set of 80 “fine” phenotypes within 32 “coarse” categories corresponding to the WBDD ontology. The median number of coarse and fine abnormalities was 12 and 18 per subject, respectively.

**Figure 2:**
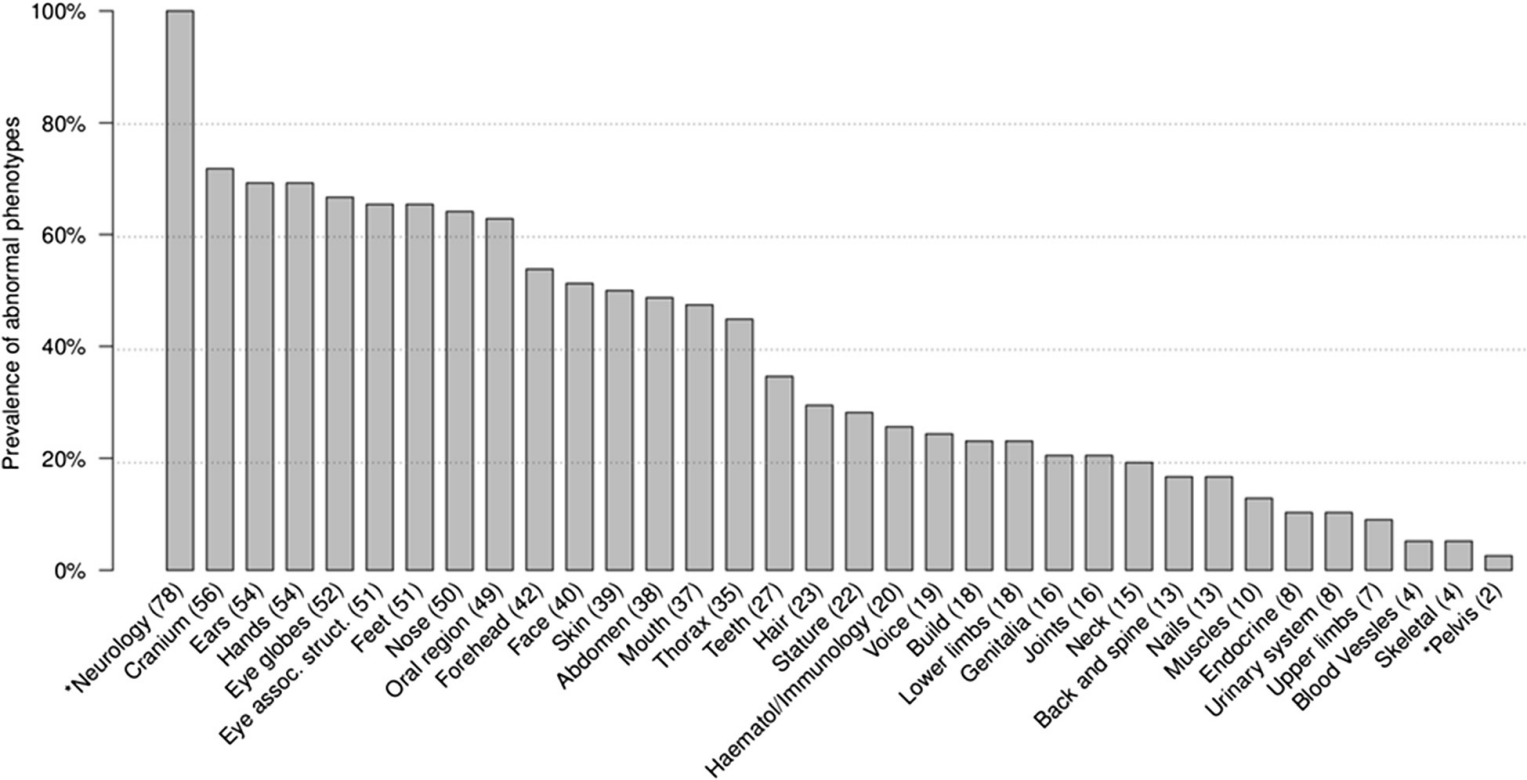
**Prevalence of abnormal coarse phenotypes.** Thirty-four coarse phenotypes were evaluated among our 78 patients based on WBDD criteria (see Additional file 1: Table S1 for the whole term of each phenotype). *indicates phenotype with >95% or <5% prevalence in the cohort which was removed in the statistical analysis.

Other than the neurology class (100%), the most prevalent phenotypes in our cohort, present in >50% of cases, were abnormalities of the head, such as abnormalities of the cranium (72%), ears (68%), eyes (67%) and nose (64%), as well as abnormalities of hands (69%) and feet (65%) (Figure 2, Additional file 1: Table S1). The median De Vries Score (Vulto-van Silfhout et al. [15]) was used to ascertain the severity of phenotypes in our cohort. Seventy-five out of 78 cases (96%) have a score ≥3 and the median De Vries score of the whole cohort is 5.

### Phenotype/genotype analysis

CNV type/phenotype data for all patients individually are presented in Additional file 1: Table S3. To explore the relationship between the abnormal phenotypes and presence of *de novo*, familial and common CNVs we examined for patients in the 3 CNV groups the median number of coarse and fine abnormalities, the modified de Vries score and the prevalence of each phenotypic feature. We also compared the median de Vries score in subjects with deletions and duplications. Finally, presence of patterns of CNV/phenotype associations for the whole cohort was explored using clustering analysis.

**Figure 3:**
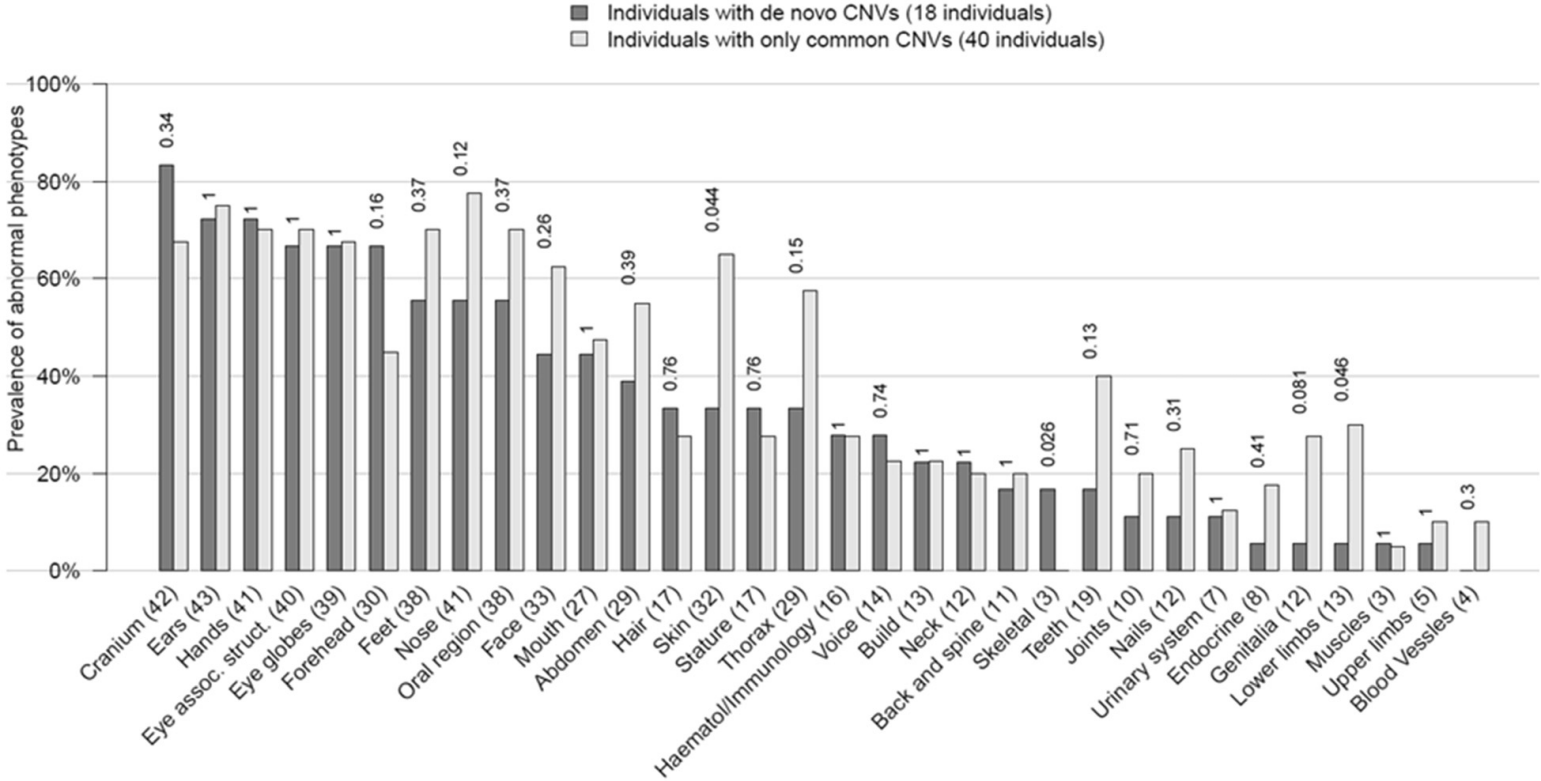
**Phenotype and *de novo *CNV association analysis.** Prevalence of the abnormality of each of the coarse phenotypes in individuals with *de novo* CNVs (18 cases) compared to individuals with only common CNVs (40 cases). The phenotypes with a prevalence >95% or <5% in the whole cohort (78 cases) were excluded from calculation.

The median number of coarse abnormalities in sub-groups of patients with de novo, familial, and common CNVs was 12.5, 10.5, and 14.5 while for fine phenotypes, it was 17.5, 14.5, and 19 for each sub-group, respectively. The modified De Vries score was 5, 4.5 and 5 for sub-groups with de novo CNVs, unique familial and common CNVs, respectively. No statistically significant difference was found for the prevalence of any of the phenotypes in different CNV groups after corrections for multiple tests (Fisher’s exact test, corrected for multiple tests). However, our data showed that among the phenotypes present in >20% of cases, abnormalities of the forehead and cranium were more prevalent in subjects with *de novo* than common CNVs (Figure 3). When 80 fine phenotypes were considered, abnormalities of forehead (i.e., shape, height, prominence etc.), of brain (structural anomalies), deafness (conductive and sensorineural) and macrocephaly (OFC >98%) were more prevalent in cases with de novo than with common CNVs, although this was not significant after multiple test corrections (Additional file 2: Figure S1).

**Figure 4:**
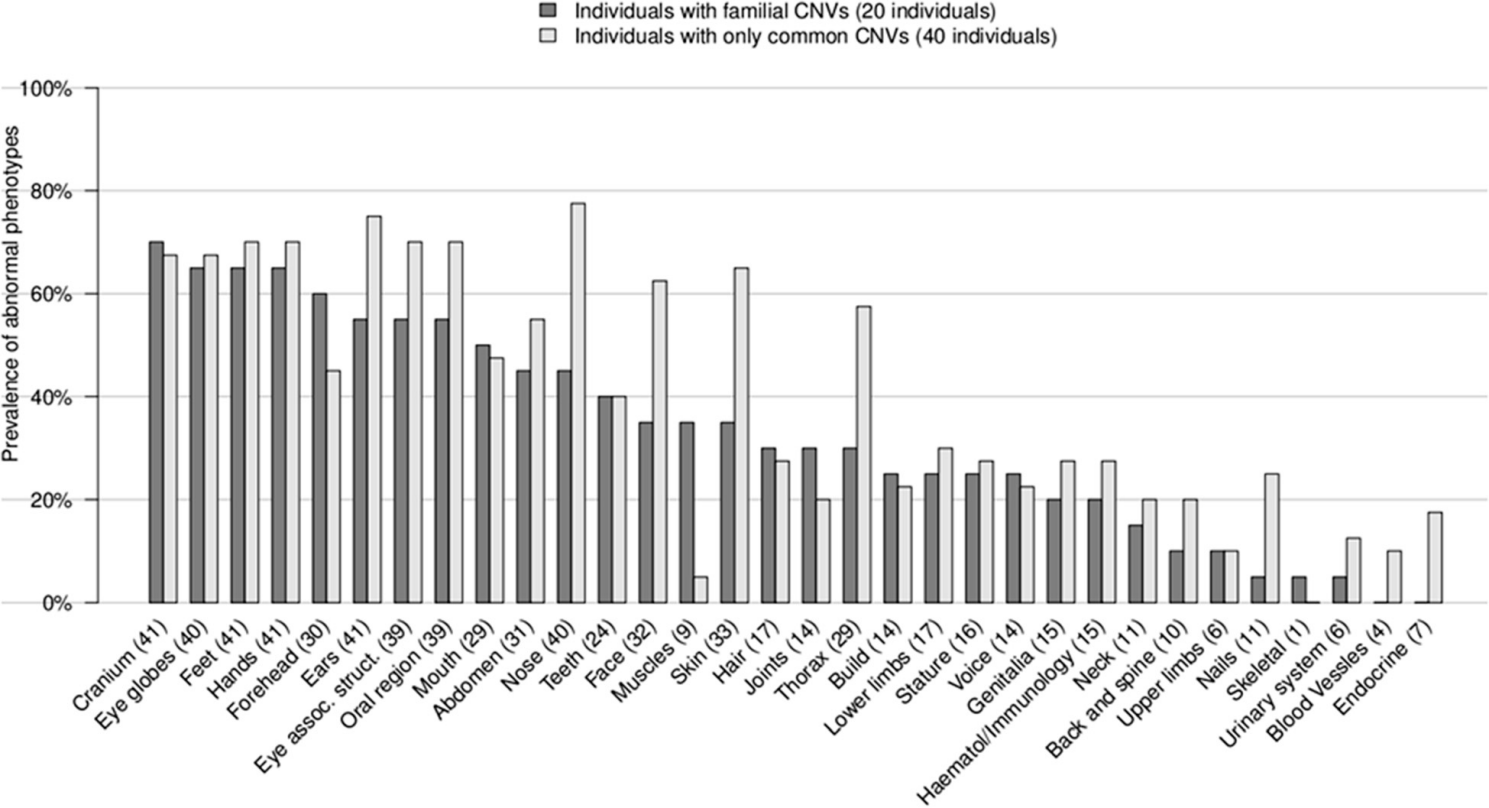
**Phenotype and familial CNV association analysis.** Prevalence of abnormal coarse phenotypes in individuals with familial CNVs (20 cases) compared with those containing only common CNVs (40 cases). Two individuals with both *de novo* and familial CNVs were removed from the analysis. The phenotypes with a prevalence >95% or <5% in the whole cohort (78 cases) were excluded from calculation.

Similarly, a higher prevalence of forehead anomalies was noted in subjects with familial CNVs when coarse phenotypes were analyzed (Figure 4). When 80 fine phenotypes were considered, number of cases with family history of ID, and with forehead anomalies was higher in the familial than common CNV group, and muscle abnormalities were seen in ~5 times more cases with familial CNVs. However, these frequencies did not reach significant levels after corrections for multiple tests (Additional file 3: Figure S2). The type of CNV (deletion or duplication) slightly affected the severity of the phenotype based on the modified De Vries score (score of 5.5 for deletions and 4.6 for duplications).

**Figure 5:**
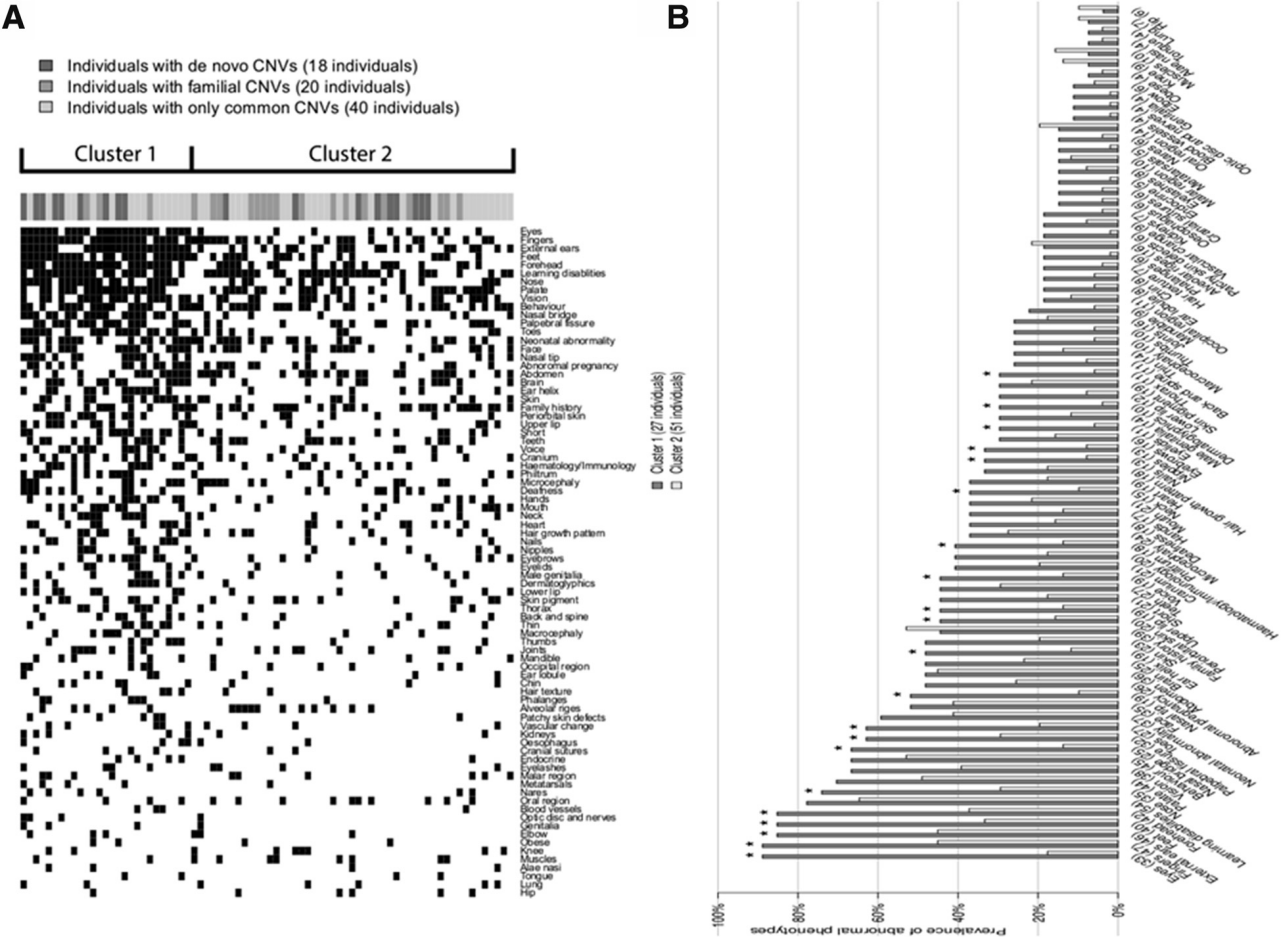
**Clustering of individuals based on 80 fine phenotypes. (A)** Data displayed as heat map. K-means method was used to group the 78 individuals into two clusters. The filled dark squares indicate an abnormal phenotype. Statistically significant differences in the number of phenotype abnormalities were found between the two clusters (P < 0.05, Wilcoxon rank-sum test). The different groups of CNVs in each individual are indicated at the top of the heat map. **(B)** Data displayed as barplot. The prevalence of individuals with an abnormal phenotype was compared between the two clusters. *indicates P < 0.05 (Fisher exact test after multiple test corrections).

Finally, to explore the association of clinical phenotypes with CNV subtypes more generally, K-means clustering analysis was performed on patients based on the 80 fine phenotypes. The optimal number of clusters was computationally determined to be two (see Methods). Individuals belonging to the first cluster had significantly more phenotypic abnormalities (mean 28.3/subject) than those from the second cluster (mean 13.5/subject; p = 2.7 × 10^-12^; Wilcoxon rank-sum test) (Figure 5). 24 out of 80 phenotypes were significantly more prevalent in cluster 1 compared to cluster 2 (P < 0.05, Fisher’s exact test after multiple test correction) (Figure 5 and Additional file 1: Table S4). We stress that differences in phenotypes between the clusters are expected since the clustering is based on the phenotypes. However, neither the number of total CNVs, the number of de novo or familial CNVs, nor CNV size segregated with the clusters.

## Discussion

This report contributes a unique exploration of the association of detailed phenotypic categories applied from the LDD with *de novo*, familial and common CNV subtypes, to systematically record, compare and report primary and secondary phenotypic abnormalities in 78 ID subjects. Our cohort consisted of subjects with a high number of phenotypic abnormalities with a median of 12 for primary and 18 for secondary features/subject. This was also reflected in a high median modified De Vries score of 5 for the whole cohort. We did not detect significant prevalence for any of the phenotypes in subjects with unique *de novo* or familial CNVs in comparison to those with common CNVs only, and it is possible that the high and comparable severity of the phenotype in three CNV subgroups in our cohort eliminated the CNV impact. Nevertheless, we noted higher prevalence of several abnormalities in the unique (*de novo* and familial) CNV subgroup in comparison to the common CNV subgroup (e.g. forehead abnormalities) while in subjects with only common CNVs, abnormalities of skin and thorax were present almost 2 times more frequently than in subjects with de novo or familial CNVs.

There are very few previous studies that correlate 10 ~ 23 phenotypic features in subjects with ID with the presence or absence of submicroscopic genomic changes. No consistent results were found among these studies regarding the specific phenotypes significantly prevalent in each cohort. De Vries et al. reported a significantly higher incidence of prenatal abnormalities and positive family history of ID in children with subtelomeric abnormalities than in patients without subtelomeric defects [12], while our previous study of ASD/ID subjects [14] noted that microcephaly and severity of ID were more significantly present in cases with pathogenic CNVs in comparison to cases without pathogenic CNVs. More recently, significantly higher prevalence of heart abnormalities in ID subjects with clinically relevant CNVs or chromosome abnormalities, was noted by Shoukier et al. [13], while statistical difference in the prevalence of microcephaly and short stature was not reported between the groups. Of note, higher prevalence of macrocephaly, epilepsy and short stature was reported in subjects with pathogenic CNVs. The most recent study by Vulto-van Silfhout et al. identified facial dysmorphism, abnormal head circumference, central nervous system anomalies, heart anomalies, urogenital anomalies and modified De Vries scores ≥3 to occur at significantly higher frequency in subjects with de novo CNVs based on assessment of >5000 subjects phenotyped using HPO.

Possible reasons for discrepancy between studies include selection biases in ID subjects that had array testing (study cohorts). For example our cohort had a median de Vries score of 5, while for the cohort of Vulto-van Silfhout et al. the median score was 2. In addition, differences in the classification of CNVs exist between studies; for example Shoukier et al. included as pathogenic CNVs large scale chromosome abnormalities and syndromic and familial CNVs, while Vulto-van Silfhout excluded syndromic CNVs caused by LCRs and divided the patients based on presence of rare de novo, familial or no rare CNVs. Finally, differences in available/recorded phenotypic characteristics of patient cohorts, differences in the selection of clinical features being evaluated, or the discrepancy in the stringency or type of statistical methods used for data analysis, could be the cause of variable genotype/phenotype associations. In our study, the clinical information was obtained retrospectively and depended on the classification and description preferences of each of the participating clinical geneticists, and these also could influence the findings. Ideally, the use of a relevant and standardized ontology classification of phenotypes derived from deep phenotyping initiatives will improve phenotype/genotype analyses relevant to scientific discovery and personalized patient management of genomic causes of ID.

The WBDD database catalogues phenotypes systematically by annotation of anatomic regions and systems for the human body. Only the primary and secondary phenotype categories with more concise descriptors were used in our study, to avoid the overwhelming detail of tertiary category designations (mostly absent from patient records). The WBDD is user-friendly and easy to master, with the definition for most of the symptoms provided by the database. However, in our consideration of specific characteristics of patients with ID phenotypes, we found the database presented some limitations. For example, it does not include prenatal information, family history, severity of ID (by IQ or adaptive/functional measures), all of which could offer essential elements of the phenotype in the context of ID. Similarly, some phenotypes commonly described in practice, such as motor delays (oral, fine, gross motor), craniofacial dysmorphism, microcephaly and macrocephaly, are not listed as isolated items in primary or secondary categories. In addition, the best match for ID is neurology in the primary category, which contains three secondary features: behaviour, learning disabilities and neuro-abnormalities. The WBDD also contains an extended number of features that are rarely reported in ID within the primary categories such as pelvis, voice and skeletal system. A directly targeted, separate, and systematic ontology system for accurate and comprehensive ID phenotypic designations would be beneficial for achieving more accurate phenotype/genotype correlation and clinical translation. This system should have a detailed description of neurodevelopmental features, considering the prevalence of cranial abnormalities in our cohort.

CNVs are only one of the possible sources of genomic variation that can be pathogenic in ID [4,5]. With the advent of whole exome or genome sequencing techniques, novel sequence mutations have been found to play important role in the pathogenesis of ID in cases with or without detected pathogenic CNVs [25-28]. Our clustering analysis allowed us to group subjects in two clusters based on frequencies of abnormalities (median 28 or 13 per subject) and it will be interest to explore the mutation types and frequencies in these two groups of patients in the future. Establishing the functional consequences of gene copy number or sequence changes is also important for the assessment of their impact on the phenotype and studies addressing closer functional and phenomic linkages are becoming more common [29-33]. Efforts to use a more standardized and detailed phenotyping system in combination with array-CGH, sequencing and gene functional analysis is needed to improve our understanding of phenotype/genotype correlations and optimize their translation into accurate genetic counselling.

## Conclusions

Our study uniquely explores the association of *de novo*, familial and common CNV subtypes with detailed phenotypes categorized by a commonly used human phenome ontology database. Our cohort consisted of cases with a high median number of phenotypic abnormalities in all CNV subgroups which possibly resulted in no significant difference in the frequency of any of the studied phenotypes between the CNV sub-groups. Nevertheless, our study provides a detailed comprehensive and systematic cross-section of the frequencies of primary and secondary phenotypes in CNV sub-groups based on WBDD. We found WBDD to be user-friendly and easy to master with the definition for most of the symptoms provided by the database. Wider use of standardized and detailed phenotyping systems in combination with current whole genome analyses, including chromosome arrays and whole genome sequencing, is needed for achieving more accurate phenotype/genotype correlation and clinical translation.

## Competing interest

We declare no conflict of interest in our manuscript titled as “Copy Number Variant analysis in a deeply phenotyped cohort of individuals with Intellectual Disability (ID)”.

## Authors’ contributions

YQ performed genetic and clinical data acquisition and analysis, and drafted the manuscript; EM performed statistical and bioinformatics analyses, and drafted the manuscript; JD participated in phenotype data analysis; BM, AC, SF and FB recruited clinical cases and reviewed the manuscript; SML recruited clinical cases, supervised phenotype data analysis and reviewed the manuscript; PP supervised statistical and bioinformatics analyses, and reviewed the manuscript; ERS supervised and designed the study, helped with data interpretation, and critically revised the manuscript. All authors read and approved the final manuscript.

## Pre-publication history

The pre-publication history for this paper can be accessed here:

http://www.biomedcentral.com/1471-2350/15/82/prepub

## Supplementary Material

Additional file 1: Table S1 WBDD phenotype frequency in our cohort. Table S2 De novo and familial CNVs detected in the cohort (hg18). Table S3 Phenotype data in 78 cases with ID. Table S4. Prevalence of abnormal fine phenotypes in two clusters. Click here for file

Additional file 2: Figure S1 Prevalence of secondary phenotypes in de novo CNV group. Prevalence of abnormal fine phenotypes in individuals with de novo CNVs (18 cases) compared with those containing only common CNVs (40 cases). The phenotypes with prevalence >95% or <5% in the whole cohort (78 cases) were excluded from calculation. Click here for file

Additional file 3: Figure S2 Prevalence of secondary phenotypes in familial CNV group. Prevalence of abnormal fine phenotypes in individuals with familial CNVs (20 cases) compared with those containing only common CNVs (40 cases). Two individuals with both de novo and familial CNVs were removed from the analysis. The phenotypes with a prevalence >95% or <5% in the whole cohort (78 cases) were excluded from calculation. Click here for file
