# Supplementary material for: Copy number variants (CNVs) analysis in a deeply phenotyped cohort of individuals with intellectual disability (ID)
Source: BMC Med Genet. 2014 Jul 16;15:82. doi: 10.1186/1471-2350-15-82 (PMC4107469; doi:10.1186/1471-2350-15-82)
Supplement: Additional file 1: Table S1 — WBDD phenotype frequency in our cohort. Table S2 De novo and familial CNVs detected in the cohort (hg18). Table S3 Phenotype data in 78 cases with ID. Table S4. Prevalence of abnormal fine phenotypes in two clusters. [file 1471-2350-15-82-S1.doc]

Table S1. WBDD phenotype frequency in our cohort

| **Primary category (coarse phenotype)** | **Secondary category (fine phenotype)** | **No.(%) of cases with the phenotype abnormality**** |
| --- | --- | --- |
| **Abdomen** |  | **38(48.7%)** |
|  | **Abdomen, general abnormalities** | **36(46.2%)** |
|  | **Anus, general abnormalities*** | **2(2.6%)** |
|  | **Colon, general abnormalities*** | **0(0%)** |
|  | **Duodenum, general abnormalities*** | **0(0%)** |
|  | **Liver/biliary system, general abnormalities*** | **2(2.6%)** |
|  | **Oesophagus, general abnormalities** | **6(7.7%)** |
|  | **Pancreas (exocrine), general abnormalities*** | **1(1.3%)** |
|  | **Spleen, general abnormalities*** | **0(0%)** |
|  | **Stomach, general abnormalities*** | **2(2.6%)** |
| **Back and spine** |  | **13(16.7%)** |
|  | **Back and spine, general abnormalities** | **11(14.1%)** |
|  | **Sacrum, general abnormalities*** | **2(2.6%)** |
|  | **Vertebra, general abnormalities*** | **2(2.6%)** |
| **Blood Vessels** |  | **4(5.1%)** |
|  | **Blood vessels, general abnormalities** | **4(5.1%)** |
| **Build** |  | **18(23.1%)** |
|  | **Thin (Physical measurements-weight <3)** | **14(17.9%)** |
|  | **Obese (Physical measurements-weight >98)** | **4(5.1%)** |
| **Cranium** |  | **56(71.8%)** |
|  | **Brain, general abnormalities** | **25(32.1%)** |
|  | **Cranial bones, general abnormalities** | **0(0%)** |
|  | **Cranial sutures, general abnormalities** | **6(7.7%)** |
|  | **Cranium, general abnormalities** | **21(26.9%)** |
|  | **Microcephaly** | **25(32.1%)** |
|  | **Macrocephaly** | **10(12.8%)** |
|  | **Occipital region, general abnormalities** | **11(14.1%)** |
|  | **Scalp, general abnormalities*** | **0(0%)** |
|  | **Sella turcica, general abnormalities*** | **0(0%)** |
| **Ears** |  | **54(69.2%)** |
|  | **Anti-helix, general abnormalities*** | **2(2.6%)** |
|  | **Anti-tragus, general abnormalities*** | **1(1.3%)** |
|  | **Auditory canal, general abnormalities*** | **0(0%)** |
|  | **Deafness, general abnormalities** | **18(23.1%)** |
|  | **Ear crus, general abnormalities*** | **2(2.6%)** |
|  | **Ear helix, general abnormalities** | **19(24.4%)** |
|  | **Ear lobule, general abnormalities** | **8(10.3%)** |
|  | **External ears, general abnormalities** | **46(59%)** |
|  | **Inner ear, general abnormalities*** | **1(1.3%)** |
|  | **Mastoids, general abnormalities*** | **0(0%)** |
|  | **middle ear, general abnormalities*** | **0(0%)** |
|  | **Tragus, general abnormalities*** | **1(1.3%)** |
| **Endocrine** |  | **8(10.3%)** |
|  | **Adrenals, general abnormalities*** | **0(0%)** |
|  | **Endocrine, general abnormalities** | **5(6.4%)** |
|  | **Pancreas (endocrine), general abnormalities*** | **0(0%)** |
|  | **Parathyroids/calcium, general abnormalities*** | **1(1.3%)** |
|  | **Pituitary, general abnormalities*** | **3(3.8%)** |
|  | **Thymus, general abnormalities*** | **0(0%)** |
|  | **Thyroid, general abnormalities*** | **1(1.3%)** |
| **Eyes, Associated structures** |  | **51(65.4%)** |
|  | **Eyebrows, general abnormalities** | **16(20.5%)** |
|  | **Eyelashes, general abnormalities** | **8(10.3%)** |
|  | **Eyelids, general abnormalities** | **11(14.1%)** |
|  | **Nasolacrimal duct, general abnormalities*** | **0(0%)** |
|  | **Palpebral fissure, general abnormalities** | **32(41%)** |
|  | **Periorbital skin, general abnormalities** | **20(25.6%)** |
|  | **Tears, general abnormalities*** | **2(2.6%)** |
| **Eyes, Globes** |  | **52(66.7%)** |
|  | **Anterior chamber, general abnormalities*** | **0(0%)** |
|  | **Conunctiva, general abnormalities*** | **0(0%)** |
|  | **Cornea, general abnormalities*** | **1(1.3%)** |
|  | **Eyes, general abnormalities** | **33(42.3%)** |
|  | **Globes, general abnormalities*** | **1(1.3%)** |
|  | **Iris, general abnormalities*** | **1(1.3%)** |
|  | **Lens, general abnormalities*** | **0(0%)** |
|  | **Macula, general abnormalities*** | **0(0%)** |
|  | **Optic disc and nerves, general abnormalities** | **4(5.1%)** |
|  | **Pupil, general abnormalities*** | **1(1.3%)** |
|  | **Retina, general abnormalities*** | **2(2.6%)** |
|  | **Sclera, general abnormalities*** | **0(0%)** |
|  | **Vision, general abnormalities** | **38(48.7%)** |
|  | **Vitreous, general abnormalities*** | **0(0%)** |
| **Face** |  | **40(51.3%)** |
|  | **Cheeks, general abnormalities*** | **3(3.8%)** |
|  | **Chin, general abnormalities** | **8(10.3%)** |
|  | **Face, general abnormalities** | **35(44.9%)** |
|  | **Malar region, general abnormalities** | **10(12.8%)** |
|  | **Mandible, general abnormalities** | **9(11.5%)** |
|  | **Maxilla, general abnormalities*** | **3(3.8%)** |
| **Feet** |  | **51(65.4%)** |
|  | **Feet, general abnormalities** | **40(51.3%)** |
|  | **Hallux, general abnormalities*** | **3(3.8%)** |
|  | **Metatarsals, general abnormalities** | **5(6.4%)** |
|  | **Tarsals, general abnormalities*** | **0(0%)** |
|  | **Toes, general abnormalities** | **27(34.6%)** |
| **Forehead** |  | **42(53.8%)** |
|  | **Forehead, prominent etc., general abnormalities** | **42(53.8%)** |
| **Genitalia** |  | **16(20.5%)** |
|  | **Female genitalia, general abnormalities*** | **2(2.6%)** |
|  | **Genitalia, general abnormalities** | **4(5.1%)** |
|  | **Male genitalia, general abnormalities** | **14(17.9%)** |
| **Haematol/Immunology** |  | **20(25.6%)** |
|  | **Haematology/Immunology, general abnormalities** | **20(25.6%)** |
| **Hair** |  | **23(29.5%)** |
|  | **Hair growth pattern, general abnormalities** | **18(23.1%)** |
|  | **Hair pigmentation, general abnormalities*** | **0(0%)** |
|  | **Hair texture, general abnormalities** | **7(9%)** |
|  | **Hair, sparse etc., general abnormalities*** | **2(2.6%)** |
| **Hands** |  | **54(69.2%)** |
|  | **Carpals, general abnormalities*** | **0(0%)** |
|  | **Dermatoglyphics, general abnormalities** | **10(12.8%)** |
|  | **Fingers, general abnormalities** | **47(60.3%)** |
|  | **Hands, general abnormalities** | **17(21.8%)** |
|  | **Metacarpals, general abnormalities*** | **1(1.3%)** |
|  | **Phalanges, general abnormalities** | **6(7.7%)** |
|  | **Thumbs, general abnormalities** | **10(12.8%)** |
| **Joints** |  | **16(20.5%)** |
|  | **Joints, general abnormalities** | **16(20.5%)** |
| **Lower limbs** |  | **18(23.1%)** |
|  | **Ankle, general abnormalities*** | **2(2.6%)** |
|  | **Femur, general abnormalities*** | **2(2.6%)** |
|  | **Fibula, general abnormalities*** | **0(0%)** |
|  | **Hip, general abnormalities** | **6(7.7%)** |
|  | **Knee, general abnormalities** | **9(11.5%)** |
|  | **Lower limbs, general abnormalities*** | **2(2.6%)** |
|  | **Patella, general abnormalities*** | **0(0%)** |
|  | **Tibia, general abnormalities*** | **0(0%)** |
| **Mouth** |  | **37(47.4%)** |
|  | **Lower lip, general abnormalities** | **12(15.4%)** |
|  | **Mouth, general abnormalities** | **21(26.9%)** |
|  | **Philtrum, general abnormalities** | **18(23.1%)** |
|  | **Upper lip, general abnormalities** | **19(24.4%)** |
| **Muscles** |  | **10(12.8%)** |
|  | **Muscles, general abnormalities** | **10(12.8%)** |
| **Nails** |  | **13(16.7%)** |
|  | **Nails, general abnormalities** | **13(16.7%)** |
| **Neck** |  | **15(19.2%)** |
|  | **Neck, general abnormalities** | **15(19.2%)** |
| **Neurology*** |  | **78(100%)** |
|  | **Behaviour, general abnormalities** | **45(57.7%)** |
|  | **Learning disabilities, general abnormalities** | **54(69.2%)** |
|  | **Neuro, general abnormalities** | **77(98.7%)** |
| **Nose** |  | **50(64.1%)** |
|  | **Alae nasi, general abnormalities** | **4(5.1%)** |
|  | **Nares, general abnormalities** | **6(7.7%)** |
|  | **Nasal bridge, general abnormalities** | **25(32.1%)** |
|  | **Nasal columella, general abnormalities*** | **3(3.8%)** |
|  | **Nasal septum, general abnormalities*** | **0(0%)** |
|  | **Nasal skin/mucosa, general abnormalities*** | **0(0%)** |
|  | **Nasal tip, general abnormalities** | **19(24.4%)** |
|  | **Nose, general abnormalities** | **35(44.9%)** |
| **Oral region** |  | **49(62.8%)** |
|  | **Alveolar riges, general abnormalities** | **16(20.5%)** |
|  | **Gums, general abnormalities*** | **1(1.3%)** |
|  | **Oral region, general abnormalities** | **14(17.9%)** |
|  | **Palate, general abnormalities** | **44(56.4%)** |
|  | **Tongue, general abnormalities** | **4(5.1%)** |
| **Pelvis*** |  | **2(2.6%)** |
|  | **Acetabulum, general abnormalities*** | **0(0%)** |
|  | **Ilium, general abnormalities*** | **1(1.3%)** |
|  | **Ischium, general abnormalities*** | **0(0%)** |
|  | **Pelvis, general abnormalities*** | **0(0%)** |
|  | **Pubis, general abnormalities*** | **0(0%)** |
|  | **Sacrosciatic notch, general abnormalities*** | **1(1.3%)** |
| **Skeletal** |  | **4(5.1%)** |
|  | **Skeleton, general abnormalities*** | **3(3.8%)** |
|  | **Tubular bones, general abnormalities*** | **1(1.3%)** |
| **Skin** |  | **39(50%)** |
|  | **Patchy skin defects, general abnormalities** | **6(7.7%)** |
|  | **Skin pigment, general abnormalities** | **19(24.4%)** |
|  | **Skin tumours, general abnormalities*** | **0(0%)** |
|  | **Skin, general abnormalities** | **23(29.5%)** |
|  | **Vascular change, general abnormalities** | **9(11.5%)** |
| **Stature** |  | **22(28.2%)** |
|  | **Short (Physical measurements-height <3)** | **21(26.9%)** |
|  | **Tall (Physical measurements-height >98)*** | **1(1.3%)** |
| **Teeth** |  | **27(34.6%)** |
|  | **Teeth, general abnormalities** | **27(34.6%)** |
| **Thorax** |  | **35(44.9%)** |
|  | **Breasts, general abnormalities*** | **1(1.3%)** |
|  | **Clavicles, general abnormalities*** | **0(0%)** |
|  | **Diaphragm, general abnormalities*** | **0(0%)** |
|  | **Heart, general abnormalities** | **19(24.4%)** |
|  | **Lung, general abnormalities** | **7(9%)** |
|  | **Nipples, general abnormalities** | **13(16.7%)** |
|  | **Pectoral/shoulder girdle, general abnormalities*** | **0(0%)** |
|  | **Ribs, general abnormalities*** | **1(1.3%)** |
|  | **Scapulae, general abnormalities*** | **0(0%)** |
|  | **Shoulder shape, general abnormalities*** | **2(2.6%)** |
|  | **Thorax, general abnormalities** | **11(14.1%)** |
| **Upper limbs** |  | **7(9%)** |
|  | **Elbow, general abnormalities** | **6(7.7%)** |
|  | **Forearm, general abnormalities*** | **1(1.3%)** |
|  | **Humerus, general abnormalities*** | **0(0%)** |
|  | **Shoulder joints, general abnormalities*** | **0(0%)** |
|  | **Upper limbs, general abnormalities*** | **0(0%)** |
|  | **Wrist, general abnormalities*** | **0(0%)** |
| **Urinary system** |  | **8(10.3%)** |
|  | **Bladder, general abnormalities*** | **0(0%)** |
|  | **Kidneys, general abnormalities** | **7(9%)** |
|  | **Renal function, general abnormalities*** | **0(0%)** |
|  | **Ureters, general abnormalities*** | **2(2.6%)** |
|  | **Urethra, general abnormalities*** | **1(1.3%)** |
|  | **Urinary tract, general abnormalities*** | **2(2.6%)** |
| **Voice** |  | **19(24.4%)** |
|  | **Voice, general abnormalities** | **19(24.4%)** |

* indicates the item has >95% or <5% prevalence in the cohort

** The percentage was obtained based on the whole cohort (78 subjects)

Table S2. De novo and familial CNVs detected in the cohort (hg18).

| **Number** | **Lab.No.** | **Chr** | **Cytoband** | | **Start (bp)** | **End (bp)** | | **Size (bp)** | **CNV type** |
| --- | --- | --- | --- | --- | --- | --- | --- | --- | --- |
| Group 1: de novo CNVs | | | | | | | | | |
| 1 | 09_03*# | 1 | p36.33 | | 554278 | 6016400 | | 5462122 | dup |
| 2 | 06_01 | 1 | p36.11 | | 26575529 | 27392834 | | 817305 | del |
| 3 | 03_02* | 2 | P15-16.1 | | 55499483 | 63368196 | | 7868713 | del |
| 4 | 06_108 | 2 | p13.3 | | 72140702 | 72924626 | | 783924 | del |
| 5 | 07_21 | 2 | q23.1 | | 148595892 | 150837093 | | 2241201 | del |
| 6 | 09_52 | 3 | q27.1 | | 184990880 | 187046113 | | 2055233 | del |
| 7 | 05_05 | 5 | q14.1 | | 80370000 | 90150000 | | 9780000 | dup |
| 8 | 09_36* | 5 | q35.2 | | 175280117 | 177360462 | | 2080345 | del |
| 9 | 09_03*# | 5 | q35.3 | | 177316179 | 180645010 | | 3328831 | del |
| 10 | 05_49 | 9 | q21.13 | | 78630000 | 81090000 | | 2460000 | dup |
| 11 | 07_27 | 10 | p12.1 | | 26714221 | 29234923 | | 2520702 | del |
| 12 | 07_21 | 10 | q21.1 | | 57249135 | 58942307 | | 1693172 | del |
| 13 | 04_48 | 11 | q24.1 | | 123150000 | 128190000 | | 5040000 | dup |
| 14 | 05_40 | 11 | q24.2 | | 124276462 | 129075841 | | 4799379 | del |
| 15 | 04_48 | 11 | q24.3 | | 128250000 | 134425035 | | 6175035 | del |
| 16 | 09_22* | 16 | p11.2 | | 29563985 | 30104991 | | 541006 | del |
| 17 | 06_14 | 16 | q22.2 | | 69620645 | 73055007 | | 3434362 | del |
| 18 | 06_88* | 17 | q21.31 | | 41011330 | 41700962 | | 689632 | del |
| 19 | 08_38 | 17 | q25.3 | | 77660313 | 78154619 | | 494306 | dup |
| 20 | 05_37 | 19 | p13.3 | | 1709657 | 5057195 | | 3347538 | dup |
| 21 | 08_43 | 21 | q22.11 | | 32764124 | 33077046 | | 312922 | dup |
| Group 2: familial CNVs | | | | | | | | | |
| 1 | 07_53 | 1 | p34.1 | 45014474 | | | 45200817 | 186343 | dup |
| 2 | 08_22* | 1 | q21.1 | 144510700 | | | 146294854 | 1784154 | dup |
| 3 | 04_18* | 1 | q21.1 | 145110000 | | | 146190000 | 1080000 | del |
| 4 | 06_67 | 1 | q44 | 244230000 | | | 244530000 | 300000 | dup |
| 5 | 08_64 | 2 | p25.3 | 596200 | | | 1103115 | 506915 | dup |
| 6 | 10_03 | 2 | p16.3 | 50676345 | | | 50772152 | 95807 | del |
| 7 | 06_40 | 2 | p12 | 78428495 | | | 79496295 | 1067800 | dup |
| 8 | 08_39 | 2 | q13 | 111115515 | | | 112819206 | 1703691 | del |
| 9 | 08_26* | 2 | q37.3 | 240422341 | | | 240636515 | 214174 | dup |
| 10 | 07_79 | 8 | q21.2 | 87145370 | | | 91502986 | 4357616 | del |
| 11 | 06_118 | 9 | q21.13 | 74432437 | | | 74641177 | 208740 | dup |
| 12 | 06_48 | 10 | q21.3 | 67992625 | | | 68228665 | 236040 | del |
| 13 | 03_24 | 11 | q25 | 133845000 | | | 134449035 | 604035 | dup |
| 14 | 06_54 | 12 | q24.11 | 108003313 | | | 108061780 | 58467 | dup |
| 15 | 06_54 | 15 | q21.1 | 42843447 | | | 43520794 | 677347 | dup |
| 16 | 06_32 | 15 | q23 | 65548713 | | | 65629616 | 80903 | del |
| 17 | 06_32* | 16 | p11.2 | 29500084 | | | 30027413 | 527329 | dup |
| 18 | 04_14 | 17 | p13.1 | 10557304 | | | 11130994 | 573690 | dup |
| 19 | 10_03 | 17 | q25.3 | 77503473 | | | 78100423 | 596950 | dup |
| 20 | 09_16 | 18 | p11.23 | 7532403 | | | 9443618 | 1911215 | dup |
| 21 | 06_40 | 18 | p11.22 | 9985330 | | | 10890656 | 905326 | dup |
| 22 | 10_03 | 20 | q11.21 | 29640678 | | | 29671722 | 31044 | del |
| 23 | 06_01 | 20 | q13.12 | 41768223 | | | 41798860 | 30637 | del |
| 24 | 06_117 | 21 | q22.11 | 34648298 | | | 34821005 | 172707 | dup |
| 25 | 05_37* | X | p22.31 | 7703932 | | | 7920259 | 216327 | dup |
| 26 | 07_91 | X | p11.22 | 50842514 | | | 51096143 | 253629 | dup |
| 27 | 07_29 | X | q12 | 65732015 | | | 66158034 | 426019 | dup |

*: Overlap with Decipher syndromic region

#: Imbalance due to paternal balanced translocation between 1p36.3 and 5q35.3

Table S3. Phenotype data in 78 cases with ID.

Group A. Cases with de novo CNVs

Group B. Cases with unique familial CNVs.

Group C. Cases with normal array results.

Table S4. Prevalence of abnormal fine phenotypes in two clusters

| **Phenotype** | **Number of individuals** | | **Proportion** | | **P-value** | **Corrected P-value** |
| --- | --- | --- | --- | --- | --- | --- |
|  | Group1 (27 cases) | Group2 (51 cases) | Group1 (27 cases) | Group2 (51 cases) | P-value | Q-value |
| Eyes | 24 | 9 | 0.889 | 0.176 | 8.37e-10 | 6.7e-08 |
| Fingers | 24 | 23 | 0.889 | 0.451 | 0.000194 | 0.00258 |
| External ears | 23 | 23 | 0.852 | 0.451 | 0.000661 | 0.0055 |
| Feet | 23 | 17 | 0.852 | 0.333 | 1.29e-05 | 0.000343 |
| Forehead | 23 | 19 | 0.852 | 0.373 | 4.85e-05 | 0.00097 |
| Learning disabilities | 21 | 33 | 0.778 | 0.647 | 0.306 | 0.408 |
| Nose | 20 | 15 | 0.741 | 0.294 | 0.000263 | 0.003 |
| Palate | 19 | 25 | 0.704 | 0.49 | 0.0941 | 0.172 |
| Vision | 18 | 20 | 0.667 | 0.392 | 0.0317 | 0.0842 |
| Behaviour | 18 | 27 | 0.667 | 0.529 | 0.336 | 0.441 |
| Nasal bridge | 18 | 7 | 0.667 | 0.137 | 4.88e-06 | 0.000195 |
| Palpebral fissure | 17 | 15 | 0.63 | 0.294 | 0.00724 | 0.0341 |
| Toes | 17 | 10 | 0.63 | 0.196 | 0.000324 | 0.00324 |
| Neonatal abnormality | 16 | 21 | 0.593 | 0.412 | 0.156 | 0.249 |
| Face | 14 | 21 | 0.519 | 0.412 | 0.474 | 0.571 |
| Nasal tip | 14 | 5 | 0.519 | 0.098 | 7.71e-05 | 0.00123 |
| Abnoromal pregnancy | 13 | 13 | 0.481 | 0.255 | 0.0758 | 0.144 |
| Abdomen | 13 | 23 | 0.481 | 0.451 | 0.816 | 0.837 |
| Brain | 13 | 12 | 0.481 | 0.235 | 0.0407 | 0.0999 |
| Ear helix | 13 | 6 | 0.481 | 0.118 | 0.000687 | 0.0055 |
| Skin | 13 | 10 | 0.481 | 0.196 | 0.0174 | 0.0558 |
| Family history | 12 | 27 | 0.444 | 0.529 | 0.635 | 0.686 |
| Periorbital skin | 12 | 8 | 0.444 | 0.157 | 0.0126 | 0.048 |
| Upper lip | 12 | 7 | 0.444 | 0.137 | 0.00481 | 0.0296 |
| Short | 12 | 9 | 0.444 | 0.176 | 0.016 | 0.0558 |
| Teeth | 12 | 15 | 0.444 | 0.294 | 0.216 | 0.309 |
| Voice | 12 | 7 | 0.444 | 0.137 | 0.00481 | 0.0296 |
| Cranium | 11 | 10 | 0.407 | 0.196 | 0.0614 | 0.123 |
| Haematology/Immunology | 11 | 9 | 0.407 | 0.176 | 0.0326 | 0.0842 |
| Philtrum | 11 | 7 | 0.407 | 0.137 | 0.0108 | 0.0433 |
| Microcephaly | 10 | 14 | 0.37 | 0.275 | 0.444 | 0.546 |
| Deafness | 10 | 8 | 0.37 | 0.157 | 0.0479 | 0.101 |
| Hands | 10 | 7 | 0.37 | 0.137 | 0.0234 | 0.0693 |
| Mouth | 10 | 11 | 0.37 | 0.216 | 0.182 | 0.265 |
| Neck | 10 | 5 | 0.37 | 0.098 | 0.0061 | 0.0338 |
| Heart | 10 | 9 | 0.37 | 0.176 | 0.0944 | 0.172 |
| Hair growth pattern | 9 | 9 | 0.333 | 0.176 | 0.159 | 0.249 |
| Nails | 9 | 4 | 0.333 | 0.0784 | 0.00835 | 0.0351 |
| Nipples | 9 | 4 | 0.333 | 0.0784 | 0.00835 | 0.0351 |
| Eyebrows | 8 | 8 | 0.296 | 0.157 | 0.237 | 0.328 |
| Eyelids | 8 | 3 | 0.296 | 0.0588 | 0.00677 | 0.0338 |
| Male genitalia | 8 | 6 | 0.296 | 0.118 | 0.066 | 0.129 |
| Dermatoglyphics | 8 | 2 | 0.296 | 0.0392 | 0.00245 | 0.0178 |
| Lower lip | 8 | 4 | 0.296 | 0.0784 | 0.0189 | 0.0583 |
| Skin pigment | 8 | 11 | 0.296 | 0.216 | 0.58 | 0.663 |
| Thorax | 8 | 3 | 0.296 | 0.0588 | 0.00677 | 0.0338 |
| Back and spine | 7 | 4 | 0.259 | 0.0784 | 0.0414 | 0.0999 |
| Thin | 7 | 7 | 0.259 | 0.137 | 0.221 | 0.31 |
| Macrocephaly | 7 | 3 | 0.259 | 0.0588 | 0.0273 | 0.0753 |
| Thumbs | 7 | 3 | 0.259 | 0.0588 | 0.0273 | 0.0753 |
| Joints | 7 | 9 | 0.259 | 0.176 | 0.395 | 0.509 |
| Mandible | 6 | 3 | 0.222 | 0.0588 | 0.0573 | 0.118 |
| Occipital region | 5 | 6 | 0.185 | 0.118 | 0.499 | 0.579 |
| Ear lobule | 5 | 3 | 0.185 | 0.0588 | 0.117 | 0.191 |
| Chin | 5 | 3 | 0.185 | 0.0588 | 0.117 | 0.191 |
| Hair texture | 5 | 2 | 0.185 | 0.0392 | 0.045 | 0.0999 |
| Phalanges | 5 | 1 | 0.185 | 0.0196 | 0.0172 | 0.0558 |
| Alveolar riges | 5 | 11 | 0.185 | 0.216 | 1 | 1 |
| Patchy skin defects | 5 | 1 | 0.185 | 0.0196 | 0.0172 | 0.0558 |
| Vascular change | 5 | 4 | 0.185 | 0.0784 | 0.262 | 0.356 |
| Kidneys | 5 | 2 | 0.185 | 0.0392 | 0.045 | 0.0999 |
| Oesophagus | 4 | 2 | 0.148 | 0.0392 | 0.174 | 0.258 |
| Cranial sutures | 4 | 2 | 0.148 | 0.0392 | 0.174 | 0.258 |
| Endocrine | 4 | 1 | 0.148 | 0.0196 | 0.0462 | 0.0999 |
| Eyelashes | 4 | 4 | 0.148 | 0.0784 | 0.437 | 0.546 |
| Malar region | 4 | 6 | 0.148 | 0.118 | 0.731 | 0.769 |
| Metatarsals | 4 | 1 | 0.148 | 0.0196 | 0.0462 | 0.0999 |
| Nares | 4 | 2 | 0.148 | 0.0392 | 0.174 | 0.258 |
| Oral region | 4 | 10 | 0.148 | 0.196 | 0.76 | 0.79 |
| Blood vessels | 3 | 1 | 0.111 | 0.0196 | 0.117 | 0.191 |
| Optic disc and nerves | 3 | 1 | 0.111 | 0.0196 | 0.117 | 0.191 |
| Genitalia | 3 | 1 | 0.111 | 0.0196 | 0.117 | 0.191 |
| Elbow | 3 | 3 | 0.111 | 0.0588 | 0.412 | 0.523 |
| Obese | 2 | 2 | 0.0741 | 0.0392 | 0.606 | 0.664 |
| Knee | 2 | 7 | 0.0741 | 0.137 | 0.485 | 0.571 |
| Muscles | 2 | 8 | 0.0741 | 0.157 | 0.48 | 0.571 |
| Alae nasi | 2 | 2 | 0.0741 | 0.0392 | 0.606 | 0.664 |
| Tongue | 2 | 2 | 0.0741 | 0.0392 | 0.606 | 0.664 |
| Lung | 2 | 5 | 0.0741 | 0.098 | 1 | 1 |
| Hip | 1 | 5 | 0.037 | 0.098 | 0.658 | 0.702 |
