# Supplementary figures and images for: Copy number variants (CNVs) analysis in a deeply phenotyped cohort of individuals with intellectual disability (ID)
Source: BMC Med Genet. 2014 Jul 16;15:82. doi: 10.1186/1471-2350-15-82 (PMC4107469; doi:10.1186/1471-2350-15-82)

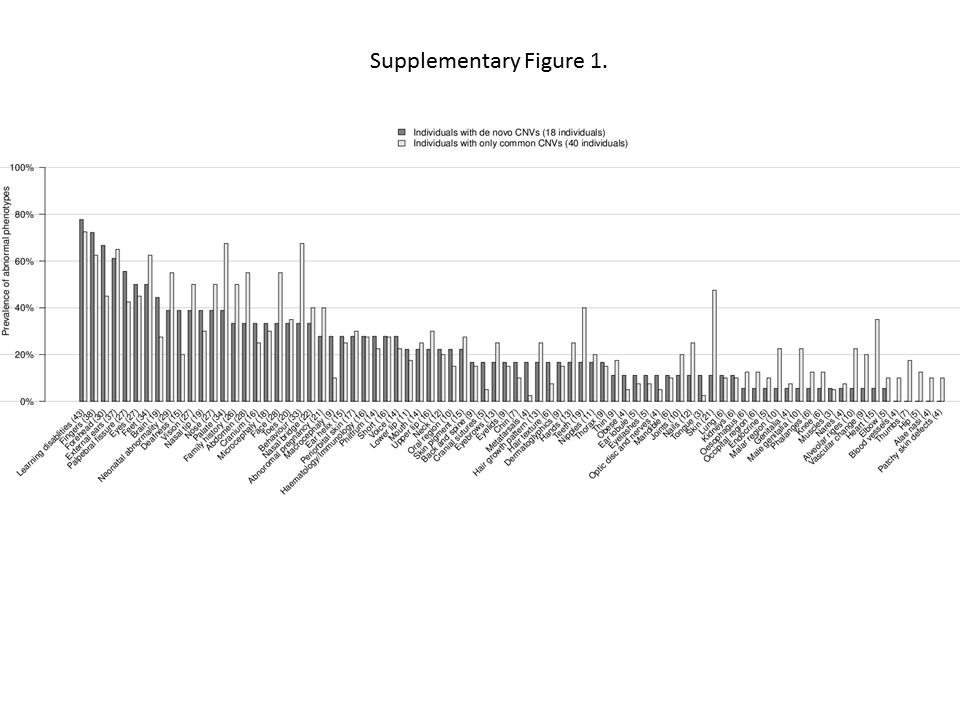

Supplement: Additional file 2: Figure S1 — Prevalence of secondary phenotypes in de novo CNV group. Prevalence of abnormal fine phenotypes in individuals with de novo CNVs (18 cases) compared with those containing only common CNVs (40 cases). The phenotypes with prevalence >95% or <5% in the whole cohort (78 cases) were excluded from calculation. [file 1471-2350-15-82-S2.tiff]

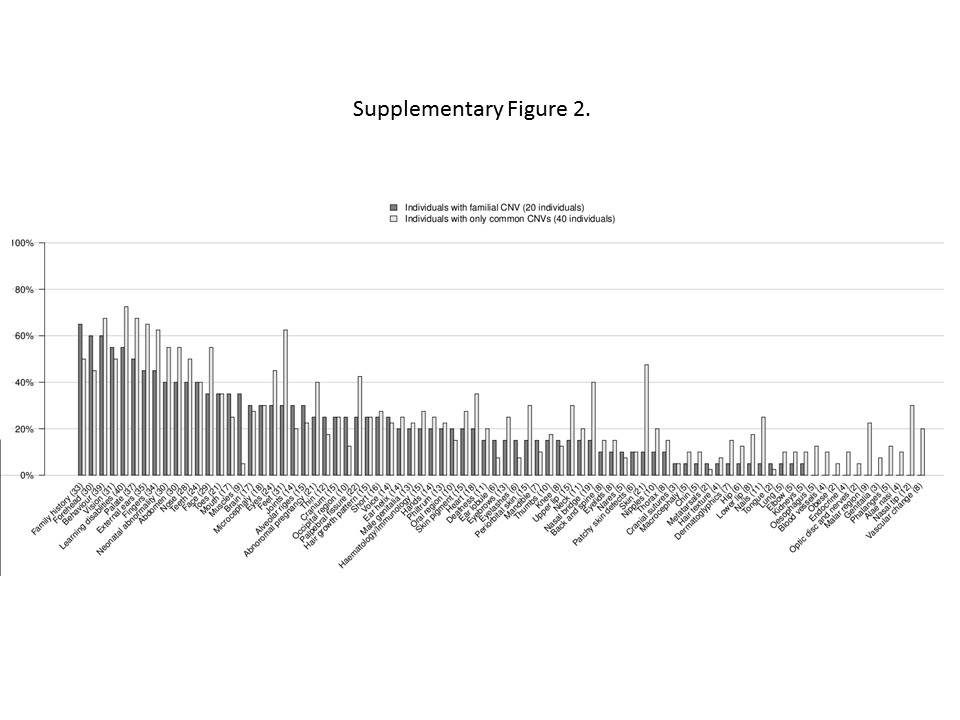

Supplement: Additional file 3: Figure S2 — Prevalence of secondary phenotypes in familial CNV group. Prevalence of abnormal fine phenotypes in individuals with familial CNVs (20 cases) compared with those containing only common CNVs (40 cases). Two individuals with both de novo and familial CNVs were removed from the analysis. The phenotypes with a prevalence >95% or <5% in the whole cohort (78 cases) were excluded from calculation. [file 1471-2350-15-82-S3.tiff]
